# *S*-Nitrosoglutathione formation at gastric pH is augmented by ascorbic acid and by the antioxidant vitamin complex, Resiston

**DOI:** 10.1080/13880209.2017.1421674

**Published:** 2018-01-03

**Authors:** Vitali I. Stsiapura, Ilya Bederman, Ivan I. Stepuro, Tatiana S. Morozkina, Stephen J. Lewis, Laura Smith, Benjamin Gaston, Nadzeya Marozkina

**Affiliations:** a Department of Biochemistry, Yanka Kupala State University, Grodno, Belarus;; b Department of Pediatrics, Case Western Reserve University, Cleveland, OH, USA;; c Department of Biochemistry, Minsk State Medical University, Minsk, Belarus;; d Divisions of Pediatrics Pulmonology, Allergy, Immunology and Sleep Medicine and Gastroenterology and Nutrition, Rainbow Babies and Children’s Hospital, Cleveland, OH, USA

**Keywords:** *S*-Nitrosylation, antioxidants, *O*-nitrosoascorbic acid

## Abstract

**Context:** Exogenous nitrogen oxides must be made bioavailable to sustain normal physiology because nitric oxide synthase (NOS) deficient mice are viable. In the stomach, *S*-nitrosoglutathione (GSNO) is formed from ingested nitrite and high levels of airway glutathione (GSH) that are cleared and swallowed. However, gastric GSNO may be broken down by nutrients like ascorbic acid (AA) before it is absorbed.

**Objective:** To study the effect of AA on GSNO formation and stability.

**Materials and methods:** GSH and nitrite were reacted with or without 5 mM AA or Resiston (5 mM AA with retinoic acid and α-tocopherol). GSNO was measured by reduction/chemiluminescence and HPLC. AA and reduced thiols were measured colorimetrically. *O*-Nitrosoascorbate and AA were measured by gas chromatography–mass spectrometry (GC–MS).

**Results:** GSNO was formed in saline and gastric samples (pH ∼4.5) from physiological levels of GSH and nitrite. Neither AA nor Resiston decreased [GSNO] at pH >3; rather, they increased [GSNO] (0.12 ± 0.19 μM without AA; 0.42 ± 0.35 μM with AA; and 0.43 ± 0.23 μM with Resiston; *n* = 4 each; *p* ≤ 0.05). However, AA compounds decreased [GSNO] at lower pH and with incubation >1 h. Mechanistically, AA, but not dehydroascorbate, increased GSNO formation; and the *O*-nitrosoascorbate intermediate was formed.

**Conclusions:** AA, with or without other antioxidants, did not deplete GSNO formed from physiological levels of GSH and nitrite at pH >3. In fact, it favoured GSNO formation, likely through *O*-nitrosoascorbate. Gastric GSNO could be a NOS-independent source of bioavailable nitrogen oxides.

## Introduction

Protein *S*-nitrosylation, the post-translational modification of a cysteine thiol by a nitric oxide (NO) group, is involved in a broad spectrum of cell signalling effects (Gow et al. 2002; Gaston, Singel, et al. 2006; Paige et al. 2008; Foster et al. 2009). In general, proteins and peptides that have been modified to form *S*-nitrosothiol bonds are involved in guanylate cyclase (GC)-independent signalling by nitrogen oxides, though *S*-nitrosylation also affects GC-dependent processes (Mayer et al. 1998). Disorders of *S*-nitrosylation are relevant to the pathophysiology of many diseases, such as cystic fibrosis, asthma, primary ciliary dyskinesia, sleep apnoea, Duchenne muscular dystrophy, etc. (Gow et al. 2002; Moya et al. 2002; Snyder et al. 2002; Gaston, Singel, et al. 2006; Colussi et al. 2008; Lim et al. 2008; Ozawa et al. 2008; Paige et al. 2008; Foster et al. 2009; Gonzalez et al. 2009; Marozkina and Gaston 2012; Marozkina et al. 2012). S-Nitrosothiols can be formed by NO synthase (NOS), by other metalloproteins, and by inorganic reactions (Mayer et al. 1998; Gow et al. 2002; Gaston, Singel, et al. 2006; Paige et al. 2008; Foster et al. 2009), but NOS knockout mice are viable (Huang 2000), suggesting that exogenous nitrogen oxides can be converted to bioavailable, physiologically sufficient nitrogen oxides. Here, we have identified a reaction in the gastric mucosa that can lead to increased formation of the endogenous, clinically beneficial *S*-nitrosothiol, *S*-nitrosoglutathione (GSNO), *in vivo*. Surprisingly, this reaction is augmented, not inhibited, by ascorbic acid (AA) at gastric pH.

GSNO was first identified in the airways (Gaston et al. 1993), but conditions would normally favour its formation in the lumen of the stomach (Gupta et al. 2016). Thus, we have focused on the reactions likely to occur in the stomach; these may also be relevant to chemistry, in the context of disease, in acidic airways and other organs (Gupta et al. 2016). Within the gastrointestinal tract, in locations other than the gastric lumen, GSNO is produced and plays a role in motility (Kiroglu et al. 2013), bile flow (Rodriguez-Ortigosa et al. 2010) and mucosal protection (Ohtake et al. 2009; Rodriguez-Ortigosa et al. 2010; Flamant et al. 2011; Savidge et al. 2011). In the proximal small bowel and ampulla of Vater, GSNO formed in the stomach will promote smooth muscle relaxation and gastric emptying (Kiroglu et al. 2013), and potentially will promote choleresis (Rodriguez-Ortigosa et al. 2010). It also has antimicrobial effects and can prevent mucosal injury (Ohtake et al. 2009; Flamant et al. 2011; Savidge et al. 2011). Importantly, GSNO absorption from the intestines appears to have systemic vascular benefits (Wu et al. 2016).

Typically, human stomach pH (3–4) is low and glutathione (GSH) levels are high. In the presence of ubiquitous nitrite, these conditions are ideal for inorganic synthesis of GSNO and other *S*-nitrosothiols (Carver et al. 2005). Airways can also have a low pH (Gaston, Kelly, et al. 2006), particularly in disease, and they also have high GSH levels; GSNO is formed (Gaston et al. 1993; Marozkina and Gaston 2015). Similar conditions exist in other organs (Gow et al. 2002; Broniowska et al. 2013), and under conditions of ischemia (Broniowska et al. 2013). GSNO exerts a range of effects relevant to host defence, ion channel regulation, smooth muscle relaxation, ciliary function and cell cycle regulation (Gaston et al. 1993; Snyder et al. 2002; Gaston, Singel, et al. 2006). High levels of GSNO formed inorganically in the stomach could augment many of these effects in the gut and/or after systemic absorption. Indeed, the GSNO catabolic enzyme, GSNO reductase, is upregulated in the stomach (Baraona et al. 2001), perhaps to prevent excessive accumulation or absorption of GSNO.

In the stomach, however, ingested nutrients produce a complex redox environment that could destabilize GSNO and related bioactive nitrogen oxides. It has been shown previously, that antioxidants such as AA can reduce GSNO to GSH and NO (Xu et al. 2000). Indeed, *S*-nitrosothiols are quite stable on the whole, and AA can, in a pH-dependent fashion, cause their rapid degradation through formation of unstable *O*-nitrosoascorbate intermediate (Aquart and Dasgupta 2004). Further, guinea-pig aorta model studies also show that AA interacts rapidly with nitrite which may result in decreased GSNO formation (Xu et al. 2000). Therefore, we hypothesized that antioxidant vitamins would lead to decreased GSNO levels with potential resulting adverse effects on GI and systemic physiology. We report, however, that AA can actually increase GSNO formation under certain physiological conditions. We tested the AA alone and AA in combination with other antioxidants (the ‘Resiston’ antioxidant complex) for their effect on the formation and stability of GSNO *in vitro* and in human gastric aspirates.

## Materials and methods

### Reagents

All reagents were purchased from Sigma-Aldrich (St. Louis, MO) unless otherwise noted.

### Synthesis of the ascorbate-NO adduct

Ascorbate-NO was made by reacting AA in citrate buffer (50 mM) with 1 µM sodium nitrite for 30 min at room temperature. Sample was lyophilized and reacted with 80 µL of bis(trimethylsilyl) trifluoroacetamide +10% trimethylchlorosilane (Regis, Morton Grove, IL) for 30 min at 75 °C. This derivatization procedure (Dintzis et al. 1995) resulted in 2,3,5,6-tetrakis-*O*-(trimethylsilyl) ether with or without –NO group.

### Chemical assays

*S*-Nitrosoglutathione was measured both by HPLC (Welch et al. 1996) and by anaerobic reduction/chemiluminescence in 1 mM cysteine saturated with CuCl as previously reported (Fang et al. 1998). Ascorbic acid was measured using the Ascorbic Acid Assay Kit (BioVision, Milpitas, CA, Catalogue #K661-100) in accordance with manufacturer’s instructions using spectrophotometry at *λ* = 570 nm (Fluostar Omega, BMG Labtech Inc, Cary, NC). Ascorbic acid and ascorbate-NO were analysed using an Agilent 5973N-MSD equipped with an Agilent 6890 GC system (GC/MS; Santa Clara, CA). A DB17-MS capillary column (30 m × 0.25 mm × 0.25 μm) was used in all assays with a helium flow of 1 mL/min. Samples were analysed in Scan mode using electron impact ionization (EI). Ion dwell time was set to 10 ms. Thiol concentrations were determined using Ellman’s reagent (dithionitrobenzoic acid) as previously described (Sedlak and Lindsay 1968). Absorbance was measured at 412 nm. Protein concentration was measured by Bradford.

### Measuring the effect of pH and time on the formation of GSNO

1. *The pH effect on GSNO formation.* Five hundred micromoles of GSH was incubated with 1 µM NaNO_2_ for 3 h at pH from 4 to 8. After pH optimization, 5 µM NaNO_2_ was incubated for 30 min in citrate buffer in presence or absence (control) 5 mM AA alone or 5 mM AA in combination with other antioxidants, Resiston. The composition of antioxidant vitamin complex Resiston is*:* vitamin A (retinol palmitate), 50,000 IU; vitamin E (α-tocopherol), 600 IU; vitamin C (AA), 2 g; β-carotenes, 20 mg. The composition has been proprietary (published Resiston drug information 2010). *S*-Nitrosothiols were detected by reduction/chemiluminescence as described previously (Fang et al. 1998).
2. *Time course.* Five hundred micromoles of GSH, 5 µM NaNO_2_ was incubated at pH 3.7 in citrate buffer in the presence or absence (control) of 5 mM AA alone or 5 mM AA in combination with other antioxidants, Resiston. *S*-Nitrosothiols were detected by reduction/chemiluminescence as above.
3. *Biological samples.* Discarded gastric aspirates (Institutional Review Board exemption) were mixed with physiological GSH 500 µM and NaNO_2_ 5 µM in the presence or absence of AA or of the clinical antioxidant complex ‘Resiston^®^’ (containing AA in a proprietary mixture with retinoic acid and α tocopherol, as previously described [published Resiston drug information 2010]); this is used as a cancer adjunctive therapy in Europe (Sukolinskii and Morozkina 1989).

### Murine lung and liver assays for the presence of ascorbate

Murine lung and liver were blanched with intravascular ice-cold PBS, then homogenized in AA assay buffer containing Ascorbic Acid Probe and Ascorbic Acid Enzyme Mix (Catalogue #K661-100 from BioVision, Milpitas, CA), centrifuged to remove cellular debris and brought to a final volume of 120 μL/well in a 96-well plate for AA assay using spectrophotometry at *λ* = 570 nm. Unreacted homogenate supernatant was used as the spectrophotometric control. Tissues were harvested in accordance with an approved IACUC protocol at the University of Virginia.

### Statistical analysis

ANOVA followed by Student's *t*-test or ANOVA on Ranks (if not parametrically distributed) was used for data analysis. All data are expressed as mean ± SD unless otherwise specified. Results with *p<* 0.05 were considered to be statistically significant.

## Results

### S-Nitrosoglutathione formation *in vitro*: effect of pH and time

**Figure 1.**
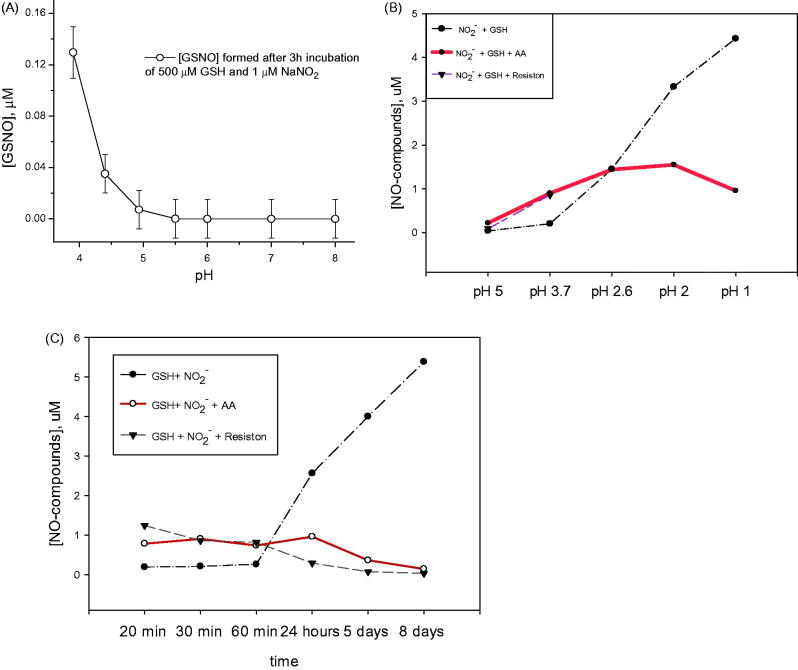
Ascorbate and an ascorbate-containing antioxidant mixture (Resiston) modify pH-dependent *S*-nitrosothiol formation. (A) GSNO formation after 3 h incubation of 500 µM GSH +1 µM NaNO_2_ was pH-dependent. *n* = 3; *p* < 0.05. (B) 500 µM GSH, 5 µM NaNO_2_ was incubated for 30 min in citrate buffer in the presence or absence (control) of 5 mM AA or Resiston. *S*-Nitrosothiols were detected by 2 °C (copper chloride cysteine) assay as described previously (Fang et al. 1998). (C) Five hundred micromoles of GSH and 5 µM NaNO_2_ was incubated at pH 3.7 in citrate buffer in the presence or absence (control) of 5 mM AA or Resiston.

Consistent with previous reports (Fang et al. 1998; Broniowska et al. 2013), GSNO formation from GSH and nitrite was pH-dependent (Figure 1(A,B)) and time-dependent (Figure 1(C)): at more basic pH, excess of OH^–^ competes with GS^–^ for NO^+^, affecting GSNO stability. As predicted (Smith and Dasgupta 2000; Xu et al. 2000), ascorbate decreased the yield of GSNO at pH <2. However, ascorbate and the ascorbate-based antioxidant actually increased GSNO formation at pH range from 3 to 5 during the first 60 min of co-incubation (Figure 1(B,C)). We hypothesized that the unstable intermediate, *O*-nitrosoascorbic acid, is produced in these conditions. Aerobic reduction/chemiluminescence method that was used for nitrosocompound detection could not completely distinguish between GSNO and *O*-nitrosoascorbate (Gow et al. 2002) and therefore the total amount of nitrosocompounds was measured; but the presence of the intermediate was confirmed by GC–MS (see below).

### S-Nitrosoglutathione formation in human stomach acid is not inhibited by ascorbic acid

Next, we aimed to determine whether the observations in Figure 1 would be relevant to the stomach environment after exposure, as in oral ingestion, to AA. We studied the formation and stability of GSNO in human gastric secretion by adding AA and antioxidant vitamin complex with AA (‘Resiston’) to saline or to *ex vivo* gastric samples. Gastric aspirates or control saline samples were mixed with 500 μM GSH and 5 μM NaNO_2_ in the presence or absence of 5 mM AA or 5 g/L of the clinical antioxidant complex Resiston (containing 5 mM AA in mixture with retinoic acid and α-tocopherol).

**Figure 2.**
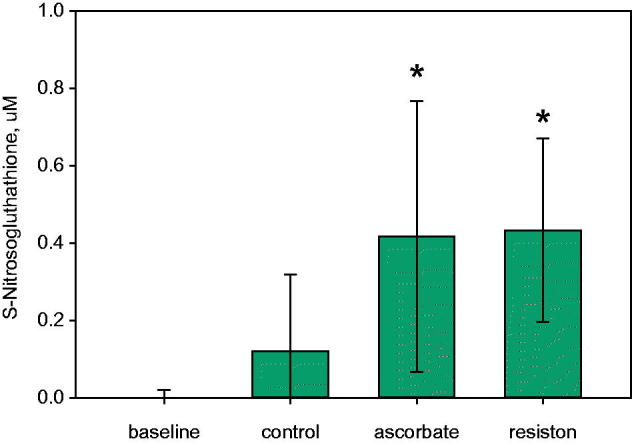
*S*-Nitrosoglutathione formation in human gastric secretions. Gastric aspirates (pH 3.5–4.5) were mixed with 500 μM GSH and 5 μM NaNO_2_ in the presence or absence of 5 mM AA or 5 mg/mL of the clinical antioxidant complex, Resiston (containing 5 mM AA in mixture with retinoic acid and tocopherol). GSNO was measured by anaerobic reduction/chemiluminescence in 1 mM cysteine saturated with CuCl as previously reported (Fang et al. 1998). *S*-nitrosothiol levels measured at baseline were *n* = 4, with addition of normal pulmonary levels of GSH and nitrite (control) (*n* = 4), ascorbate (*n* = 5, *p* ≤ 0.05) and Resiston (*n* = 4, *p* ≤ 0.05).

Two grams of AA (0.011 M) is the amount in each dose of the pharmaceutical compound Resiston. Our calculation was based on the assumption that the stomach has a volume of 1–2 L after eating and drinking. Thus, the concentration would be 5–10 mM after ingesting Resiston. GSNO was formed in saline and gastric fluid (pH 4.5) (Giorgi et al. 1989; Flagg et al. 1994; Broniowska et al. 2013; Marozkina and Gaston 2015). Levels at baseline *ex vivo* were <10 nM (*n* = 4; Figure 2). With augmentation to pulmonary levels of GSH and nitrite (recapitulating swallowed material from lung clearance [Gaston et al. 1993]), *S*-nitrosothiol levels increased to 0.121 ± 0.19 μM (*n* = 4). Surprisingly, both AA and Resiston increased GSNO levels (levels were 0.417 ± 0.35 μM and 0.433 ± 0.23 μM, respectively, *n* = 4 each, *p* ≤ 0.05). A 3–4-fold increase of GSNO production in the presence of AA or Resiston was observed within 1 h of incubation. However, GSNO was lost at pH <3 and at incubation time >60 min (Figure 1(B,C)).

### Ascorbate is normally present in lungs and other tissues

**Figure 3.**
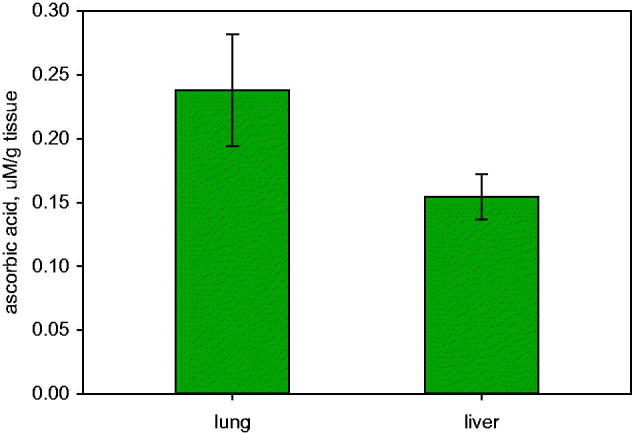
Ascorbic acid is normally present in lungs and liver. Ascorbic acid in murine lung (*n* = 10) and liver (*n* = 10).

To determine whether endogenous AA levels were also high enough to contribute to GSNO stability, we measured concentrations in the murine lung and liver homogenates. The baseline AA level detected in the mouse liver was 0.154 ± 0.017 µM/g tissue (*n* = 10) and in the lungs, 0.238 ± 0.043 µM/g tissue (*n* = 10) (Figure 3). Concentrations were lower than AA concentrations detected in mouse lungs and liver by other researches, though the AA concentrations in tissues are highly dependent on diet (Kratzing and Kelly 1982; Reidling et al. 2008; Iwama et al. 2012). These concentrations are not optimal to augment GSNO formation. This suggests that it may be a unique circumstance in the stomach, or under conditions in which the pH is low in the airways, gut and elsewhere (Benjamin et al. 1994; Ng et al. 2004; Hunt 2007) that leads to the augmented nitrosothiol formation.

### Ascorbate does not increase GSNO in mild acid by increasing the levels of reduced thiol substrate

**Figure 4.**
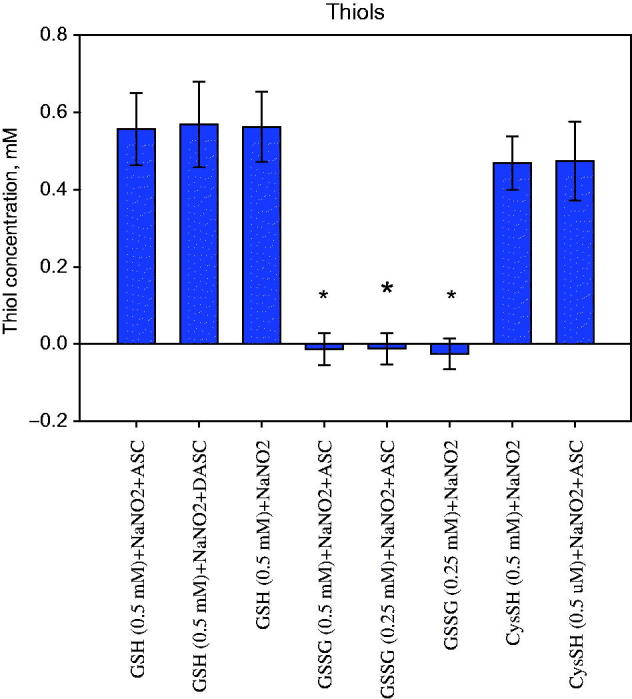
Ascorbate is not acting to keep thiols reduced in the presence of nitrite. Amount of thiols was detected by Ellman’s reagent (Thermo Scientific, #22582, Waltham, MA) in the presence of 5 µM NaNO_2_ and 5 mM ascorbic or 5 mM dehydroascorbic acid (*n* = 4, *p ≤ 0.001 compared to all groups with reduced thiols).

**Figure 5.**
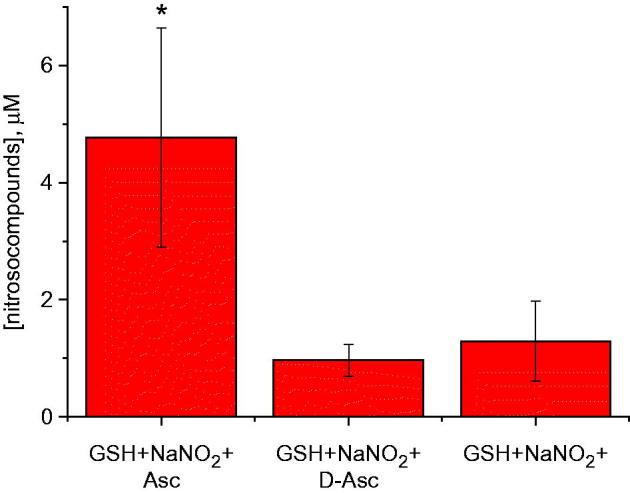
*S*-Nitrosoglutathione formation *in vitro* can be augmented by ascorbic acid (but not dehydroascorbic acid) at low pH. Five micromoles of sodium nitrite was incubated with 5 mM AA or 5 mM dehydroascorbic acid in the presence of 0.5 mM GSH at pH 3.7 for 1 h. Nitrosocompounds concentration was measured by anaerobic reduction/chemiluminescence in 1 mM cysteine saturated with CuCl as previously reported (Fang et al. 1998) (*n* = 3), *p < 0.05.

To determine whether the effect of AA to augment GSNO was because AA reduced GSSG to form GSH, we performed the reaction shown in Figure 5 in the presence of reduced and oxidized thiols. AA did not increase concentrations of reactant thiol (Figure 4).

**Figure 6.**
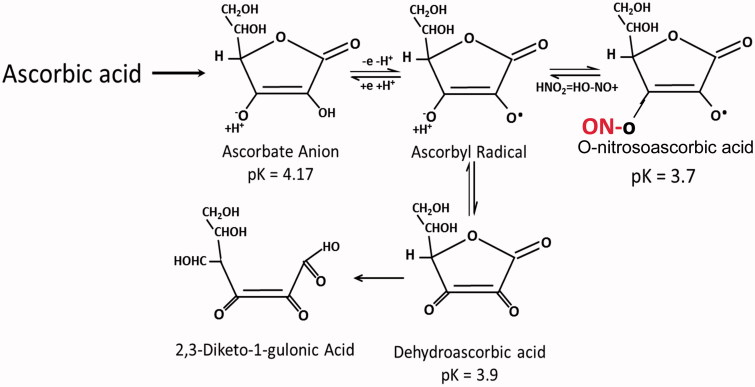
Proposed scheme. Ascorbic acid makes *O*-nitrosoascorbate, an intermediate containing NO^+^ group at pH 3.7. Ascorbate anion exists in equilibrium with ascorbyl radical. NO^+^ can be attached to the oxygen anion of ascorbyl radical at pH =3.7. Alternatively, ascorbyl radical can acquire electron and form dehydroascorbic acid that can be converted to 2,3-diketo-1-gulonic acid.

**Figure 7.**
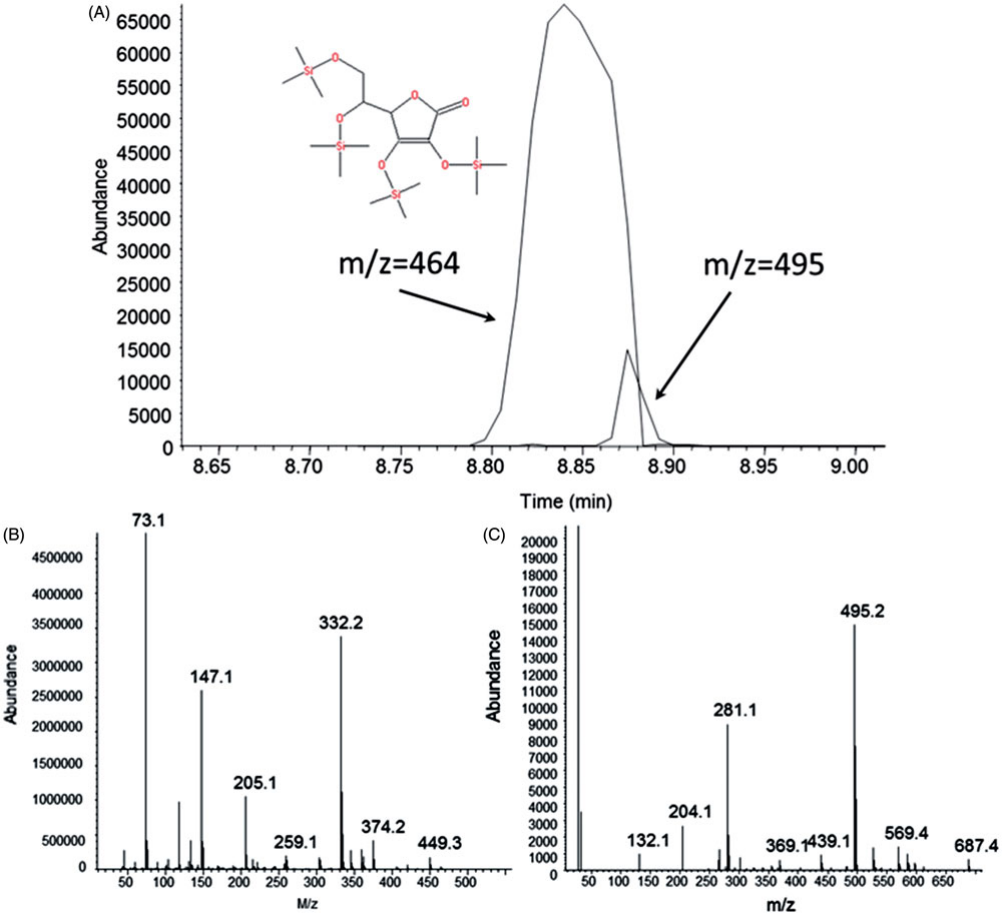
(Panel A) Gas-chromatography of ascorbic acid 2,3,5,6-tetrakis-*O*-(trimethylsilyl) ether derivative and its nitro adduct. Ascorbic acid derivative (M 464) was formed as addition of TMS_4_ (288) to ascorbic acid MW (176). Nitro adduct (M + 31) is represented by small peak (M 495). (Panel B) Mass spectrum of ascorbic acid (molecular ion with methyl group loss (M–15), *m*/*z* = 449). (Panel C) Mass spectrum of ascorbic acid nitro adduct (molecular ion (M + 35) *m*/*z* = 495).

To get insight of the mechanism behind augmented yield of nitrosocompounds in the presence of AA, we studied whether oxidized AA (dehydroascorbate) affects nitrosocompounds formation. We observed that, at pH 3.7, dehydroascorbate did not have pronounced effect on increase of nitrosocompound concentration in the presence of GSH and nitrite over one hour (Figure 5). This suggests that the active intermediate, ascorbyl radical, could stabilize NO^+^ (Figure 6). We therefore hypothesized that ascorbate increased the total amount of produced nitrosocompounds due to capture of nitrosonium NO^+^ by AA (or ascorbate) with formation of relatively stable compound *O*-nitrosoascorbic acid (or *O*-nitrosoascorbate) (Figure 6). *O*-Nitrosoascorbate is a short-lived, intermediate product of AA oxidation to dehydroascorbic acid (Figure 7) and apparently serves here as an NO^+^ donor to make GSNO. On the other hand, GSNO when formed, has a *T*_1/2_ of 10 h at pH 3 of 24 h (Nikitovic and Holmgren 1996). We acknowledge that some of our signal (Figure 2) may result from detection of unstable *O*-nitroascorbate, but this appears to be a minor and transient intermediate (Figure 7; also, see below).

### Ascorbic acid is modified by acidified nitrite

To add an NO group to form the nitrosoascorbate intermediate, we have reacted nitrite with ascorbate (concentrations as above) at pH 3.7 for 1 h. Addition of NO group to AA was verified using gas chromatography–mass spectrometry (Figure 7). From comparison of the peak areas, we found about 7% of the product with *m*/*z* 495 which is consistent with formation of *O*-nitrosoascorbyl radical from parent mass of 464 (M + 31).

## Conclusions and discussion

We conclude that strong biological reducing agents in the form of AA and antioxidant vitamins do not deplete the beneficial GSNO formed in human gastric contents at physiological gastric pH; in fact, they can augment it via transnitrosation from *O*-nitrosoascorbic acid to GSH. Dissociation energies of O–NO bonds are lower in comparison with S–NO bonds (Wu et al. 2016) and one can expect preferable GSNO formation in transnitrosation reactions. This suggests that GSNO formed in the stomach could be a source of bioavailable nitrogen oxides in the human gut under normal conditions (Ohtake et al. 2009; Rodriguez-Ortigosa et al. 2010; Flamant et al. 2011; Savidge et al. 2011; Kiroglu et al. 2013; Wu et al. 2016). Nitrite alone is reduced at low pH to NO radical, which can be inactivated by haemoglobin (Marozkina and Gaston 2015). *S*-Nitrosothiols, once formed, can transport bioactive nitrogen oxides in a regulated fashion to target tissues (Gupta et al. 2016; Wu et al. 2016). However, our data also suggest that this chemistry may be primarily observed in the stomach because other tissues have high pH and/or low ascorbate concentration. An exception could be the inflamed lung under the conditions of high ascorbate ingestion/levels (Flagg et al. 1994) and low pH (Gaston, Kelly, et al. 2006).

Our data suggest that the increased GSNO formation at pH range of 2.5–4.5 in the presence of ascorbate could most likely be explained by *O*-nitrosoascorbic acid formation that serves as a donor–acceptor for NO^+^ (Figure 7). The AA does not appear to act through reduction of oxidized thiol (Figure 4). With regard to the possibility that GSNO could be formed in the stomach, our data confirm that GSNO is formed from physiological levels of GSH and nitrite in the aqueous solutions at different pH and in human gastric fluid. Reaction of nitrous acid HNO_2_ with GSH is responsible for the GSNO formation and its rate increases with lower pH due to the protonation of nitrite ion:

(NO_2_^-^+H^+^↔HNO_2_ with pK_a_ ∼3.4)

$$\text{GSH}+{\text{HNO}}_{2}↔\text{GSH}+{\text{NO}}^{+}-{\text{OH}}^{-}↔\text{GSNO}+{\text{H}}_{2}\text{O}$$

One can see from Figure 1 that the GSNO formation in mixtures of GSH and nitrite can be detected at pH <5. In accordance with this result, GSNO was formed in human gastric fluid (pH∼3.5) from physiological levels of GSH and nitrite (Figure 2). We believe that Resiston was superior to nitrosylated compound formation to AA alone in the short time frame, 20 min (Figure 2(C)), but Resiston and AA were equal in the longer period of time for nitrosylated compound formation (Figure 1(C)). We think that change in pH made Resiston and AA alone equal to each other in term of formation of nitrosylated compound (Figure 1(A,B)). Both AA and Resiston significantly increased nitrosylated compound formation in gastric contents (Figure 2) compared to control (GSH and nitrite was added to the gastric contents) and baseline (gastric contents alone), but neither AA nor Resiston was superior to each other in this process. Note that even higher concentrations of AA might not be effective nitrosylated compound formation because of possible oxidative (pro-oxidant) properties of very high concentrations of the AA (Seo and Lee 2002).

In addition to the question of GSNO formation in human gastric secretions, we also addressed the stability of GSNO in this environment and whether this source of periodic GSNO formation (from swallowed GSH) can be physiologically relevant. *S*-Nitrosothiols can be reduced in the presence of electron donors:

(*) $$\text{GSNO}+{\text{H}}^{+}+{\text{e}}^{-}↔\text{NO}+\text{GSH}$$

Reduction potential for reaction (*) was found to be *E*_r_ = –0.98 V at pH 7.4 (Ford et al. 2002) and there are several reducing agents that can contribute in GSNO degradation process. For example, redox couple Cu^+^/Cu^2+^ with reduction potential *E*_0_ = 0.16 V.

Cu^2+^(aq)+e^–^ → Cu^+^(aq), and, of note, free Cu^2+^ is present in the proximal mammalian gut (Flamant et al. 2011). Additionally, Cu^+^ ions can react with molecular oxygen producing superoxide anions and hydroxyl radicals that may also contribute in GSNO degradation.

Here, we have focused on the effect of AA on the GSNO concentration because it is an essential vitamin and reducing agent present in many foods, and is used ubiquitously in the nutraceutical industry as an orally ingested electron donor (‘nutritional antioxidant’). It is well known that presence of AA promotes decomposition of GSNO and other *S*-nitrosothiols at physiological pH; this reaction is widely used to measure *S*-nitrosothiol concentrations (Paige et al. 2008). Reaction of AA with *S*-nitrosothiols was studied earlier (Holmes and Williams 2000; Paige et al. 2008; Melzer et al. 2012) and two reaction pathways were identified. The first one dominates at low concentration of AA and is Cu^2+^ dependent and the second route becomes important at higher concentrations of AA and is [Cu^2+^]-independent. The first mechanism involves reduction of Cu^2+^ ions by AA to form Cu^+^ which further donates electron to GSNO resulting in NO and GSH production.

$$2{\text{Cu}}^{2+}+\text{AA}\to 2{\text{Cu}}^{+}+\text{ DASC}$$

$${\text{Cu}}^{+}+\text{GSNO }+{\text{ H}}^{+}\to {\text{Cu}}^{2+}+\text{ GSH }+\text{ NO}$$

At higher AA concentrations or in the presence of Cu^2+^ chelators, the reaction proceeds via intermediate step of *O*-nitrosoascorbate formation in transnitrosation process yielding NO or NO^–^ (Figure 6)

$$\text{GSNO }+{\text{ ASC}}^{-}\to {\text{ASCNO}}^{-}\;+{\text{ GS}}^{-}\;\left(\text{n}\right)$$

$${\text{ASCNO}}^{-}\to {\text{ASC}}^{-}\;+\text{ NO }\left(\text{x}\right)$$

$${\text{ASCNO}}^{-}\to \text{DASC }+{\text{ NO}}^{-}\;\left(\text{y}\right)$$

Rates of reactions (x), (y) and therefore the yield ratio of [NO^–^]/[NO] were found to depend on pH with dominant formation of NO^–^ at physiological conditions. Rate of *O*-nitrosoascorbate formation showed dramatic dependence on pH, decreasing by two orders of magnitude when pH changed from 7.3 to 3.6 (Holmes and Williams 2000). This demonstrates that at low pH values degradation of GSNO by AA is less efficient. Moreover, AA can directly react with HNO_2_ producing *O*-nitrosoascorbate, i.e., this reaction can be a source of *O*-nitroso compounds (NO^+^ centred on O-atom of AA) in addition to *S*-nitroso compounds.

In basic pH, excess OH^–^ competes with NO^+^ and HONO is deprotonated to form nitrite: GSNO formation and stability are impaired. In acidic pH, the balance is reversed: HONO is stabilized, and NO + transfer to GS– is favoured: OH– is the leaving group, which is depleted to form water to drive the reaction towards GSNO. However, HONO can also evolve NO is: NO loss competes with GSNO formation. Our data suggest that 2-nitrosyl ascorbyl, perhaps in equilibrium with 2-nitrosyl ascorbate, stabilizes NO^+^, favouring transfer to GSH. Functionally, GSNO is favoured, though additional mechanistic work will be needed.

This work is important because high oral doses of AA and AA-containing antioxidant complexes are commonly used and have benefits in a number of diseases (Cameron and Pauling 1978; Sukolinskii and Morozkina 1989; Morozkina et al. 1991; Du et al. 2012; Rodrigo et al. 2014). Our data suggest that, rather than depleting gastric GSNO levels as we expected, these high exposure levels stabilize GSNO at gastric pH. This could actually augment gastric motility, vascular smooth muscle relaxation and host defence. Additional studies will be required, however, to determine the fate of GSNO under these conditions, and whether GSNO contributes to the beneficial effects of antioxidant complexes. The gastric chemistry of nitrogen oxides is also important because nitric oxide synthase (NOS) deficient mice are viable (Huang 2000). We have hypothesized that exogenous nitrogen oxides in the gut and lung can provide nitrogen oxides in a NOS-independent fashion. For example, the lung lining fluid normally contains near mM levels of reduced GSH and μM levels of nitrite; these are cleared from the airways to the larynx and swallowed. Under conditions of mild acidification in the gut and distal airways, inorganic formation of luminal *S*-nitrosoglutathione (GSNO) would be predicted (Gaston et al. 1993; Fang et al. 1998; Gaston, Singel, et al. 2006; Marozkina and Gaston 2012, 2015). Our current data suggest that this NOS-independent source of stable nitrogen oxide bioactivity is stabilized, rather than broken down, by oral ingestion of AA given as an antioxidant therapy.

## References

[CIT0001] Aquart DV, Dasgupta TP. 2004 Dynamics of interaction of vitamin C with some potent nitrovasodilators, *S*-nitroso-*N*-acetyl-d,l-penicillamine (SNAP) and *S*-nitrosocaptopril (SNOCap), in aqueous solution. Biophys Chem. 107:117–131. 14962594 10.1016/j.bpc.2003.08.011

[CIT0002] Baraona E, Abittan CS, Dohmen K, Moretti M, Pozzato G, Chayes ZW, Schaefer C, Lieber CS. 2001 Gender differences in pharmacokinetics of alcohol. Alcohol Clin Exp Res. 25:502–507. 11329488

[CIT0003] Benjamin N, O'Driscoll F, Dougall H, Duncan C, Smith L, Golden M, McKenzie H. 1994 Stomach NO synthesis. Nature. 368:502. 10.1038/368502a0 8139683

[CIT0004] Broniowska KA, Diers AR, Hogg N. 2013 *S*-nitrosoglutathione. Biochim Biophys Acta. 1830:3173–3181. 23416062 10.1016/j.bbagen.2013.02.004 PMC3679660

[CIT0005] Cameron E, Pauling L. 1978 Supplemental ascorbate in the supportive treatment of cancer: reevaluation of prolongation of survival times in terminal human cancer. Proc Natl Acad Sci USA. 75:4538–4542. 279931 10.1073/pnas.75.9.4538 PMC336151

[CIT0006] Carver J, Doctor A, Zaman K, Gaston B. 2005 *S*-nitrosothiol formation. Meth Enzymol. 396:95–105. 16291225 10.1016/S0076-6879(05)96010-2

[CIT0007] Colussi C, Mozzetta C, Gurtner A, Illi B, Rosati J, Straino S, Ragone G, Pescatori M, Zaccagnini G, Antonini A, et al 2008 HDAC2 blockade by nitric oxide and histone deacetylase inhibitors reveals a common target in Duchenne muscular dystrophy treatment. Proc Natl Acad Sci USA. 105:19183–19187. 19047631 10.1073/pnas.0805514105 PMC2614736

[CIT0008] Dintzis FR, Laszlo JA, Nelsen TC, Baker FL, Calvert CC. 1995 Free and total ion concentrations in pig digesta. J Anim Sci. 73:1138–1146. 7628958 10.2527/1995.7341138x

[CIT0009] Du J, Cullen JJ, Buettner GR. 2012 Ascorbic acid: chemistry, biology and the treatment of cancer. Biochim Biophys Acta. 1826:443–457. 22728050 10.1016/j.bbcan.2012.06.003 PMC3608474

[CIT0010] Fang K, Ragsdale NV, Carey RM, MacDonald T, Gaston B. 1998 Reductive assays for *S*-nitrosothiols: implications for measurements in biological systems. Biochem Biophys Res Commun. 252:535–540. 9837741 10.1006/bbrc.1998.9688

[CIT0011] Flagg EW, Coates RJ, Eley JW, Jones DP, Gunter EW, Byers TE, Block GS, Greenberg RS. 1994 Dietary glutathione intake in humans and the relationship between intake and plasma total glutathione level. Nutr Cancer. 21:33–46. 8183721 10.1080/01635589409514302

[CIT0012] Flamant M, Aubert P, Rolli-Derkinderen M, Bourreille A, Neunlist MR, Mahe MM, Meurette G, Marteyn B, Savidge T, Galmiche JP, et al 2011 Enteric glia protect against *Shigella flexneri* invasion in intestinal epithelial cells: a role for *S*-nitrosoglutathione. Gut. 60:473–484. 21139062 10.1136/gut.2010.229237

[CIT0013] Ford E, Hughes MN, Wardman P. 2002 The reaction of superoxide radicals with *S*-nitrosoglutathione and the products of its reductive heterolysis. J Biol Chem. 277:2430–2436. 11709557 10.1074/jbc.M109310200

[CIT0014] Foster MW, Hess DT, Stamler JS. 2009 Protein *S*-nitrosylation in health and disease: a current perspective. Trends Mol Med. 15:391–404. 19726230 10.1016/j.molmed.2009.06.007 PMC3106339

[CIT0015] Gaston B, Kelly R, Urban P, Liu L, Henderson EM, Doctor A, Teague WG, Fitzpatrick A, Erzurum S, Hunt JF. 2006 Buffering airway acid decreases exhaled nitric oxide in asthma. J Allergy Clin Immunol. 118:817–822. 17030232 10.1016/j.jaci.2006.06.040

[CIT0016] Gaston B, Reilly J, Drazen JM, Fackler J, Ramdev P, Arnelle D, Mullins ME, Sugarbaker DJ, Chee C, Singel DJ, et al 1993 Endogenous nitrogen oxides and bronchodilator *S*-nitrosothiols in human airways. Proc Natl Acad Sci USA. 90:10957–10961. 8248198 10.1073/pnas.90.23.10957 PMC47900

[CIT0017] Gaston B, Singel D, Doctor A, Stamler JS. 2006 *S*-nitrosothiol signaling in respiratory biology. Am J Respir Crit Care Med. 173:1186–1193. 16528016 10.1164/rccm.200510-1584PP PMC2662966

[CIT0018] Giorgi G, Micheli L, Segre G, Pecchi A. 1989 Glutathione (GSH) in human stomach mucosa. Riv Eur Sci Med Farmacol. 11:163–167. 2799001

[CIT0019] Gonzalez DR, Treuer A, Sun QA, Stamler JS, Hare JM. 2009 *S*-Nitrosylation of cardiac ion channels. J Cardiovasc Pharmacol. 54:188–195. 19687749 10.1097/FJC.0b013e3181b72c9f PMC3390783

[CIT0020] Gow AJ, Chen Q, Hess DT, Day BJ, Ischiropoulos H, Stamler JS. 2002 Basal and stimulated protein *S*-nitrosylation in multiple cell types and tissues. J Biol Chem. 277:9637–9640. 11796706 10.1074/jbc.C100746200

[CIT0021] Gupta P, Yakubov S, Tin K, Zea D, Garankina O, Ghitan M, Chapnick EK, Homel P, Lin YS, Koegel MM. 2016 Does alkaline colonic pH predispose to *Clostridium difficile* infection? South Med J. 109:91–96. 26840963 10.14423/SMJ.0000000000000414

[CIT0022] Holmes AJ, Williams DLH. 2000 Reaction of ascorbic acid with *S*-nitrosothiols: clear evidence for two distinct reaction pathways. J Chem Soc Perkin Trans. 2:1639–1644.

[CIT0023] Huang PL. 2000 Mouse models of nitric oxide synthase deficiency. J Am Soc Nephrol. 11 Suppl 16:S120–S123. 11065342

[CIT0024] Hunt J. 2007 Exhaled breath condensate pH assays. Immunol Allergy Clin N Am. 27:597–606. 10.1016/j.iac.2007.09.006 PMC2858452 17996578

[CIT0025] Iwama M, Amano A, Shimokado K, Maruyama N, Ishigami A. 2012 Ascorbic acid levels in various tissues, plasma and urine of mice during aging. J Nutr Sci Vitaminol. 58:169–174. 22878386 10.3177/jnsv.58.169

[CIT0026] Kiroglu OE, Aydinoglu F, Ogulener N. 2013 The effects of thiol modulators on nitrergic nerve- and *S*-nitrosothiols-induced relaxation in duodenum. J Basic Clin Physiol Pharmacol. 24:143–150. 23412870 10.1515/jbcpp-2012-0054

[CIT0027] Kratzing CC, Kelly JD. 1982 Tissue levels of ascorbic acid during rat gestation. Int J Vitam Nutr Res. 52:326–332. 7174230

[CIT0028] Lim KH, Ancrile BB, Kashatus DF, Counter CM. 2008 Tumour maintenance is mediated by eNOS. Nature. 452:646–649. 18344980 10.1038/nature06778 PMC2688829

[CIT0029] Marozkina NV, Gaston B. 2012 *S*-Nitrosylation signaling regulates cellular protein interactions. Biochim Biophys Acta. 1820:722–729. 21745537 10.1016/j.bbagen.2011.06.017 PMC4035225

[CIT0030] Marozkina NV, Gaston B. 2015 Nitrogen chemistry and lung physiology. Annu Rev Physiol. 77:431–452. 25668023 10.1146/annurev-physiol-021113-170352

[CIT0031] Marozkina NV, Wei C, Yemen S, Wallrabe H, Nagji AS, Liu L, Morozkina T, Jones DR, Gaston B. 2012 *S*-nitrosoglutathione reductase in human lung cancer. Am J Respir Cell Mol Biol. 46:63–70. 21816964 10.1165/rcmb.2011-0147OC PMC3262657

[CIT0032] Mayer B, Pfeiffer S, Schrammel A, Koesling D, Schmidt K, Brunner F. 1998 A new pathway of nitric oxide/cyclic GMP signaling involving *S*-nitrosoglutathione. J Biol Chem. 273:3264–3270. 9452441 10.1074/jbc.273.6.3264

[CIT0033] Medical News (Russian). 2010. Something seems missing here published Resiston drug information.

[CIT0034] Melzer MM, Mossin S, Cardenas AJ, Williams KD, Zhang S, Meyer K, Warren TH. 2012 A copper(II) thiolate from reductive cleavage of an *S*-nitrosothiol. Inorg Chem. 51:8658–8660. 22867516 10.1021/ic301356h

[CIT0035] Morozkina TS, Sukolinskii VN, Strel'nikov AV. 1991 The selective effect of vitamin E, A, and C complexes on antioxidant protection in neoplastic and normal tissues. Vopr Med Khim. 37:59–61. 1812616

[CIT0036] Moya MP, Gow AJ, Califf RM, Goldberg RN, Stamler JS. 2002 Inhaled ethyl nitrite gas for persistent pulmonary hypertension of the newborn. Lancet. 360:141–143. 12126827 10.1016/S0140-6736(02)09385-6

[CIT0037] Ng AW, Bidani A, Heming TA. 2004 Innate host defense of the lung: effects of lung-lining fluid pH. Lung. 182:297–317. 15742242 10.1007/s00408-004-2511-6

[CIT0038] Nikitovic D, Holmgren A. 1996 *S*-nitrosoglutathione is cleaved by the thioredoxin system with liberation of glutathione and redox regulating nitric oxide. J Biol Chem. 271:19180–19185. 8702596 10.1074/jbc.271.32.19180

[CIT0039] Ohtake K, Shimada N, Uchida H, Kobayashi J. 2009 Proteomic approach for identification of protein *S*-nitrosation in mouse gastric mucosa treated with *S*-nitrosoglutathione. J Proteomics. 72:750–760. 19278666 10.1016/j.jprot.2009.03.001

[CIT0040] Ozawa K, Whalen EJ, Nelson CD, Mu Y, Hess DT, Lefkowitz RJ, Stamler JS. 2008 *S*-nitrosylation of beta-arrestin regulates beta-adrenergic receptor trafficking. Mol Cell. 31:395–405. 18691971 10.1016/j.molcel.2008.05.024 PMC2630185

[CIT0041] Paige JS, Xu G, Stancevic B, Jaffrey SR. 2008 Nitrosothiol reactivity profiling identifies *S*-nitrosylated proteins with unexpected stability. Chem Biol. 15:1307–1316. 19101475 10.1016/j.chembiol.2008.10.013 PMC2628636

[CIT0042] Reidling JC, Subramanian VS, Dahhan T, Sadat M, Said HM. 2008 Mechanisms and regulation of vitamin C uptake: studies of the hSVCT systems in human liver epithelial cells. Am J Physiol Gastrointest Liver Physiol. 295:G1217–G1227. 18845575 10.1152/ajpgi.90399.2008 PMC2604802

[CIT0043] Rodrigo R, Hasson D, Prieto JC, Dussaillant G, Ramos C, Leon L, Garate J, Valls N, Gormaz JG. 2014 The effectiveness of antioxidant vitamins C and E in reducing myocardial infarct size in patients subjected to percutaneous coronary angioplasty (PREVEC Trial): study protocol for a pilot randomized double-blind controlled trial. Trials. 15:192. 24885600 10.1186/1745-6215-15-192 PMC4050098

[CIT0044] Rodriguez-Ortigosa CM, Banales JM, Olivas I, Uriarte I, Marin JJ, Corrales FJ, Medina JF, Prieto J. 2010 Biliary secretion of *S*-nitrosoglutathione is involved in the hypercholeresis induced by ursodeoxycholic acid in the normal rat. Hepatology. 52:667–677. 20683964 10.1002/hep.23709

[CIT0045] Savidge TC, Urvil P, Oezguen N, Ali K, Choudhury A, Acharya V, Pinchuk I, Torres AG, English RD, Wiktorowicz JE, et al 2011 Host *S*-nitrosylation inhibits clostridial small molecule-activated glucosylating toxins. Nat Med. 17:1136–1141. 21857653 10.1038/nm.2405 PMC3277400

[CIT0046] Sedlak J, Lindsay RH. 1968 Estimation of total, protein-bound, and nonprotein sulfhydryl groups in tissue with Ellman's reagent. Anal Biochem. 25:192–205. 4973948 10.1016/0003-2697(68)90092-4

[CIT0047] Seo MY, Lee SM. 2002 Protective effect of low dose of ascorbic acid on hepatobiliary function in hepatic ischemia/reperfusion in rats. J Hepatol. 36:72–77. 11804667 10.1016/s0168-8278(01)00236-7

[CIT0048] Smith JN, Dasgupta TP. 2000 Kinetics and mechanism of the decomposition of *S*-nitrosoglutathione by l-ascorbic acid and copper ions in aqueous solution to produce nitric oxide. Nitric Oxide. 4:57–66. 10733873 10.1006/niox.2000.0272

[CIT0049] Snyder AH, McPherson ME, Hunt JF, Johnson M, Stamler JS, Gaston B. 2002 Acute effects of aerosolized *S*-nitrosoglutathione in cystic fibrosis. Am J Respir Crit Care Med. 165:922–926. 11934715 10.1164/ajrccm.165.7.2105032

[CIT0050] Sukolinskii VN, Morozkina TS. 1989 Prevention of postoperative complications in patients with stomach cancer using an antioxidant complex. Voprosy Onkol. 35:1242–1245. 2596070

[CIT0051] Welch GN, Upchurch GR Jr, Loscalzo J. 1996 *S*-nitrosothiol detection. Meth Enzymol. 268:293–298. 8782595 10.1016/s0076-6879(96)68031-8

[CIT0052] Wu W, Perrin-Sarrado C, Ming H, Lartaud I, Maincent P, Hu XM, Sapin-Minet A, Gaucher C. 2016 Polymer nanocomposites enhance *S*-nitrosoglutathione intestinal absorption and promote the formation of releasable nitric oxide stores in rat aorta. Nanomed: Nanotechnol Biol Med. 12:1795–1803. 10.1016/j.nano.2016.05.006 27184095

[CIT0053] Xu A, Vita JA, Keaney JF Jr. 2000 Ascorbic acid and glutathione modulate the biological activity of *S*-nitrosoglutathione. Hypertension. 36:291–295. 10948092 10.1161/01.hyp.36.2.291

